# Retinoic acid regulates cell-shape and -death of E-FABP (FABP5)-immunoreactive septoclasts in the growth plate cartilage of mice

**DOI:** 10.1007/s00418-017-1578-0

**Published:** 2017-05-12

**Authors:** Yasuhiko Bando, Miyuki Yamamoto, Koji Sakiyama, Hide Sakashita, Fuyoko Taira, Genki Miyake, Shoichi Iseki, Yuji Owada, Osamu Amano

**Affiliations:** 1 Division of Anatomy, Meikai University School of Dentistry, 1-1 Keyakidai, Sakado, Saitama 3500283 Japan; 2 Department of Histology and Embryology, Kanazawa University Graduate School of Medical Sciences, 13-1 Takara-machi, Kanazawa, Ishikawa 9208640 Japan; 3 Division of Maxillofacial Surgery II, Meikai University School of Dentistry, 1-1 Keyakidai, Sakado, Saitama 3500283 Japan; 4 Department of Organ Anatomy, Tohoku University Graduate School of Medicine, 2-1 Seiryo-machi, Aoba-ku, Sendai, Miyagi 9808575 Japan

**Keywords:** Septoclast, E-FABP, Retinoic acid, Immunohistochemistry, Growth plate, Mouse

## Abstract

Septoclasts, which are mononuclear and spindle-shaped cells with many processes, have been considered to resorb the transverse septa of the growth plate (GP) cartilage at the chondro-osseous junction (COJ). We previously reported the expression of epidermal-type fatty acid-binding protein (E-FABP, FABP5) and localization of peroxisome proliferator-activated receptor (PPAR)β/δ, which mediates the cell survival or proliferation, in septoclasts. On the other hand, retinoic acid (RA) can bind to E-FABP and is stored abundantly in the GP cartilage. From these information, it is possible to hypothesize that RA in the GP is incorporated into septoclasts during the cartilage resorption and regulates the growth and/or death of septoclasts. To clarify the mechanism of the cartilage resorption induced by RA, we administered an overdose of RA or its precursor vitamin A (VA)-deficient diet to young mice. In mice of both RA excess and VA deficiency, septoclasts decreased in the number and cell size in association with shorter and lesser processes than those in normal mice, suggesting a substantial suppression of resorption by septoclasts in the GP cartilage. Lack of PPARβ/δ-expression, TUNEL reaction, RA receptor (RAR)β, and cellular retinoic acid-binding protein (CRABP)-II were induced in E-FABP-positive septoclasts under RA excess, suggesting the growth arrest/cell-death of septoclasts, whereas cartilage-derived retinoic acid-sensitive protein (CD-RAP) inducing the cell growth arrest or morphological changes was induced in septoclasts under VA deficiency. These results support and do not conflict with our hypothesis, suggesting that endogenous RA in the GP is possibly incorporated in septoclasts and utilized to regulate the activity of septoclasts resorbing the GP cartilage.

## Introduction

Septoclasts, previously termed perivascular cells, are located at the chondro-osseous junction (COJ) between the growth plate (GP) cartilage and the metaphysis (Schenk et al. 1967; Lee et al. 1995; Nakamura and Ozawa 1996). The cells extend thin and long processes toward the transverse septa comprising uncalcified cartilage matrices in the GP. Enzymes working in degradation of the uncalcified cartilage matrix such as cathepsin B (Lee et al. 1995) and matrix metalloproteinase (MMP)-13 (Nakamura et al. 2004) have been detected in septoclasts. Histological and biochemical features above have suggested that septoclasts resorb the uncalcified cartilage matrices at the GP (Lee et al. 1995).

We previously reported exclusive expression and localization of epidermal-type fatty acid-binding protein (E-FABP, FABP5) in septoclasts in the GP of mice (Bando et al. 2014). Fatty acid-binding proteins (FABPs) bind long-chain fatty acids or other hydrophobic molecules and transport them intracellularly. Several types of FABP, such as liver type (L-FABP, FABP1), heart type (H-FABP, FABP3), and brain type (B-FABP, FABP7), have been identified and their functions are tissue-specific (Storch and Thumser 2010). E-FABP was originally found in psoriatic keratinocytes (Madsen et al. 1992) and has been revealed to be responsible for the water permeability barrier of the skin (Owada et al. 2002). E-FABP binds to saturated fatty acids or some types of polyunsaturated fatty acids (Hanhoff et al. 2002), and it translocates from the cytosol to the nucleus to interact with peroxisome proliferator-activated receptor (PPAR)β/δ, resulting in induction of the cell differentiation by enhancement of PPARβ/δ-mediated transcriptional activity (Tan et al. 2002). E-FABP-positive septoclasts are simultaneously immunoreactive for PPARβ/δ (Bando et al. 2014). Cartilage matrices of the GP contain abundant amounts of fatty acids (Havivi and Bernstein 1969); therefore, they are incorporated into septoclasts containing FABP during the cartilage resorption. Fatty acids are transported by E-FABP from the cytosol into mitochondria to be involved in the energy metabolism via β-oxidation (Bando et al. 2014).

In addition to these fatty acids, retinoic acid (RA), a biologically active metabolite of vitamin A (VA), can bind to E-FABP (Schug et al. 2007). RA transported by E-FABP activates PPARβ/δ, resulting in enhanced cell survival/proliferation, while RA transported by cellular retinoic acid-binding protein (CRABP)-II activates retinoic acid receptor (RAR), resulting in cell growth arrest or apoptosis (Schug et al. 2007; Pavone et al. 2010). RA is synthesized from VA supplied by retinyl esters or carotenoids in dietary components (Ross 1993; Al Tanoury et al. 2013). VA is essential for the embryonic development, epithelial differentiation, immune function, and bone formation as well as the vision (reviewed by Ross et al. 2000). The previous studies have shown that RA at an extremely high or low concentration induces morphological changes and growth inhibition of cells and tissues. In cultured cells, excessive RA induces reduction in cell size, changes in cell-shape (Seewaldt et al. 1995; Lee et al. 2012), and growth inhibition or apoptosis mediated by RARβ (Seewaldt et al. 1995; Si et al. 1996; Koszinowski et al. 2015). Under low RA concentration, the expression of cartilage-derived retinoic acid-sensitive protein (CD-RAP), alternatively referred to as melanoma inhibitory activity (MIA), was observed at high levels in melanoma cell lines or cartilaginous cells (Dietz and Sandell 1996). CD-RAP is a cell growth inhibitory factor and its expression is accompanied by morphological changes, resulting in a compact and spherical cell-shape (Blesch et al. 1994).

Endogenous RA exists in the hypertrophic zone of the GP in a sufficient amount to elicit the retinoic signaling and action (Williams et al. 2010). The RA in the hypertrophic zone of the GP is, therefore, taken by cartilage-resorbing cells. This issue leads us to hypothesize that RA incorporated into septoclasts during the cartilage resorption regulates septoclastic growth and/or death. To clarify the mechanism of the cartilage resorption induced by RA, the present study was attempted to investigate whether or not the excess of RA or deficiency of VA influences on the cell-shape, number, and death of septoclasts in the GP of mouse tibiae.

## Materials and methods

### Animals

Male ddY mice were used in the present study, and they were purchased from Sankyo Labo Service (Tokyo, Japan). Mice under RA excess (*n* = 10): All-trans retinoic acid of 300 mg/Kg (Sigma-Aldrich, St. Louis, MO, USA) in soybean oil was administrated to 4-week-old (P4w) mice by oral gavage, while only a vehicle was administered to control mice (*n* = 10). Mice were sacrificed and fixed 2 days after the retinoic acid or vehicle injection (De Luca et al. 2000).

Mice under VA deficiency (*n* = 10): Mice at P3w were fed with a VA-free diet for 6 weeks according to the established method (Wolbach 1947). Vitamin A-free diet was prepared from the AIN-93G diet (Oriental Yeast, Tokyo, Japan). Normal control mice (*n* = 10) were fed a standard AIN-93G diet (Oriental Yeast) for the same period. All the mice were sacrificed and fixed at P9w.

### Tissue preparation

Mice were administered with pentobarbital sodium (30-mg/kg body weight) and perfused through the heart first with saline and subsequently with 4% paraformaldehyde in 0.1-M phosphate buffer, pH 7.4. Then, the tibiae were dissected and immersed in the same fixative overnight at 4 °C, and subsequently decalcified in 0.5-M ethylenediaminetetraacetic acid (EDTA, pH 7.2) for 20 days at 4 °C. Specimens were then immersed overnight in 30% sucrose in 0.1-M phosphate buffer. Proximal tibiae were dissected out from the knee joints, and frozen sagittal sections of 10-μm thickness were cut on a cryostat. Thin sections of 20-μm thickness were used for three-dimensional (3D) morphological analyses.

### Measurement of the height of the growth plate

To measure the height of the GP, we performed a morphometrical analysis. Midsagittal sections of the proximal tibiae collected from ten specimens for each group were stained routinely with toluidine blue. The height was measured in the middle of the GP. Fifty sections for each group were measured and analyzed statistically.

### Immunohistochemistry of E-FABP

Cryosections on glass slides were treated for 60 min in 0.3% Triton X-100 in phosphate-buffered saline (PBS), and then in 10% fetal bovine serum (FBS, Nichirei, Tokyo, Japan) in 0.1-M PBS for 60 min. After rinse with PBS, the sections were treated overnight at room temperature with rabbit anti-rat E-FABP polyclonal antibody (0.5 μg/ml, Owada et al. 2001) in PBS. After another rinsing with PBS, they were incubated for 60 min with the secondary antibody, i.e., biotin-conjugated goat anti-rabbit IgG (426012; Nichirei). After further rinse, they were reacted for 45 min with horseradish peroxidase (HRP)-conjugated streptavidin (Nichirei). After final rinse with PBS, 3,3′-diaminobenzidine tetrahydrochloride (DAB, Dojindo, Kumamoto, Japan) was applied to visualize sites of the antigen–antibody reaction.

### Double immunofluorescence staining

To clarify factors inducing changes in cell-shape and/or decrease in number of E-FABP-positive septoclasts under RA excess or VA deficiency, we performed double immunofluorescent staining for E-FABP plus PPARβ/δ, a mediator of cell proliferation induced by RA (Schug et al. 2007), LC3, a marker for autophagy (Tanida et al. 2004), RARβ, a mediator of RA-induced apoptosis (Seewaldt et al. 1995; Koszinowski et al. 2015), or CD-RAP (MIA), cell growth inhibitory factor under low RA-concentrated condition (Blesch et al. 1994; Dietz and Sandell 1996).

Sections were treated with a mixture of rabbit anti-E-FABP antibody (0.5 μg/ml in PBS) and goat anti-mouse PPARβ/δ (sc-1987; 1:40, Santa Cruz Biotechnology, Santa Cruz, CA, USA) or goat anti-human MAP LC3 α/β (sc-16756; 1:50, Santa Cruz Biotechnology) or goat anti-human RARβ (sc-552-G; 1:50, Santa Cruz Biotechnology) or goat anti-human MIA (sc-17048; 1:50, Santa Cruz Biotechnology), and a mixture of rabbit anti-human CRABP-II (10225-1-AP; 1:50, Proteintech, Chicago, IL, USA) and goat anti-human cathepsin B (AF965; 10 μg/ml, R&D Systems, Minneapolis, MN, USA) in PBS. Goat anti-cathepsin B antibody was used as a marker for septoclasts (Lee et al. 1995) instead of rabbit anti-E-FABP antibody (Bando et al. 2014) to perform double immunostaining with rabbit anti-CRABP-II antibody, based on our previous finding that E-FABP and cathepsin B were co-localized in septoclasts (Bando et al. 2014). After rinse with PBS, the sections were treated with a mixture of FITC-conjugated donkey anti-rabbit IgG (AP182F; 1:200, Merck Millipore, Billerica, MA, USA) plus Cy3-conjugated donkey anti-goat IgG (AP180C; 1:200, Merck Millipore). Observations were made by use of a conventional light microscope or a confocal laser-scanning microscope (LSM5-Exciter, Carl Zeiss, Obercohen, Germany).

### TUNEL reaction

To detect apoptosis, TUNEL reaction was applied. For double immunostaining for E-FABP and TUNEL reaction, sections were first treated overnight at room temperature with rabbit anti-E-FABP antibody (0.5 μg/ml in PBS). Thereafter, fragmented DNA by apoptosis was labeled with digoxigenin using Apoptag Plus Fluorescein In Situ Apoptosis Detection Kit (S7111; Chemicon, Temecula, CA, USA). After rinse with PBS, sections were treated with a mixture of Cy3-conjugated donkey anti-rabbit IgG (AP182C; 1:200, Merck Millipore) plus fluorescein-conjugated sheep anti-digoxigenin antibody (included in the Detection Kit). Observations were made under a confocal laser-scanning microscope.

### Cell counts

The number of septoclasts per area was measured according to our previous study (Bando et al. 2014) by taking an average of the numbers of E-FABP-immunoreactive cell bodies in unit squares with 200 μm × 1000 μm along the line of the COJ. In the present study, a septoclast with a clearly observable cell body containing a nucleus was counted as a cell. Ten squares collected from ten specimens for each group were measured and analyzed statistically. To examine the apoptotic induction in septoclasts under RA excess, simultaneously E-FABP-immunoreactive and TUNEL-positive apoptotic cells were counted at the COJ by the same method.

### Three-dimensional analysis

Sections of 20-μm thickness were stained in immunofluorescence with anti-E-FABP antibody (0.5 μg/ml) and Cy3-conjugated donkey anti-rabbit IgG (AP182C; 1:200, Merck Millipore). Sections were subsequently treated with 4,6-diamidino-2-phenylindole dihydrochloride (DAPI; ImmunoBioScience, Mukilteo, WA, USA) for nuclear staining and observed using a confocal laser-scanning microscope (LSM5-Exciter, Carl Zeiss). 3D images were reconstructed by use of Zen 2008 software (Carl Zeiss).

### Electron microscopic immunohistochemistry

For electron microscopic immunohistochemistry, the pre-embedding immunoreaction method was performed according to our previous studies (Amano et al. 2001; Yamamoto et al. 2016). Briefly, the immunostained sections with anti-E-FABP antibody were postfixed with 0.5% OsO_4_ for 20 min. After block-staining with 1% uranyl acetate for 30 min, the sections were dehydrated by passage through a graded ethanol series and embedded in Glicidether 100 (Selva Feinbiochemica, Heidelberg, Germany). Ultrathin sections were prepared and observed with a Hitachi H-7650 electron microscope (Tokyo, Japan).

### Statistical analysis

The cell counts were expressed as the mean ± standard deviation, and they were treated by means of Scheffe’s multiple comparison test. *P* values <0.01 were regarded as statistically significant.

## Results

### Influence by RA excess or VA deficiency

**Fig. 1:**
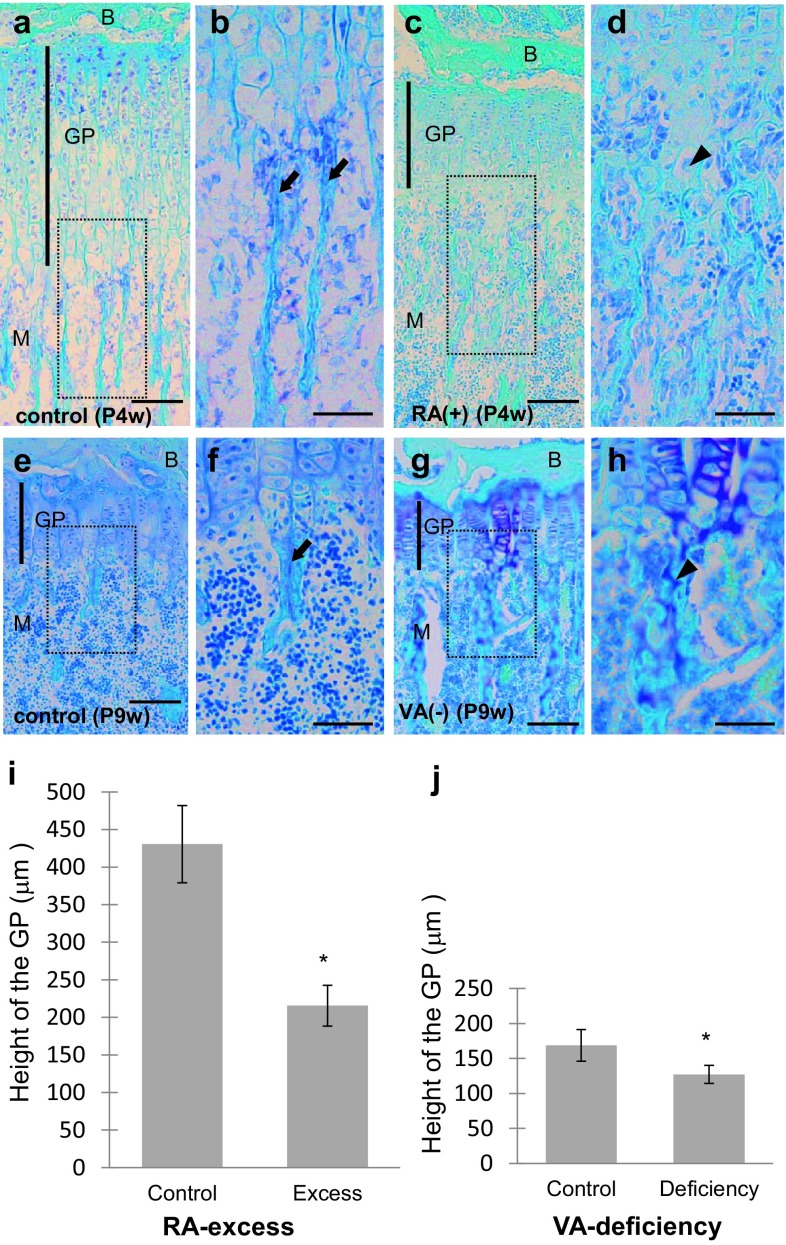
The growth plate (GP) in mouse tibiae. Light micrographs of toluidine blue-stained sections at lower (**a**, **c**, **e**, **g**) and higher (**b**, **d**, **f**, **h** *squares* in **a**, **c**, **e**, and **g**, respectively) magnifications and graphs (**i**, **j**) showing the effects of retinoic acid (RA)-excessive administration for 4-week-old (P4w) (**c**, **d**; **a**, **b**: control) or vitamin A (VA)-deficient diet (**g**, **h**, 6 weeks from P3w; **e**, **f**: control) on the GP of the proximal tibiae of mice. *B* epiphyseal bone, *M* metaphysis, *arrows* trabeculae, *arrowheads* cartilage, *scale bars* 100 μm; mean ± SD; *P < 0.01 (*n* = 50)

Histology and morphometrical analyses by RA excess and VA deficiency were shown in Fig. 1. When compared with the normal control mice at the same age (Fig. 1a, b, e, f), the GP in the proximal tibiae was thinner in both mice administered with RA excess and those with VA deficiency (Fig. 1c, d, g, h). The height of the GP decreased in RA excess by approximately 50% of the control (Fig. 1i). The height of the GP also decreased in VA deficiency by approximately 25% of the control (Fig. 1j). These findings were consistent with the previous ones (De Luca et al. 2000; Wolbach 1947), and showed that the administration of RA excess and VA deficiency was successfully performed in the present study.

In addition, abnormally thick bone trabeculae including vertically arranged chondrocytes were observed in the GP in RA excess (Fig. 1c, d) and VA deficiency (Fig. 1g, h). In control mice, such abnormal trabeculae were not observed (Fig. 1a, b, e, f).

### Changes in shape and number of E-FABP-immunopositive septoclasts

**Fig. 2:**
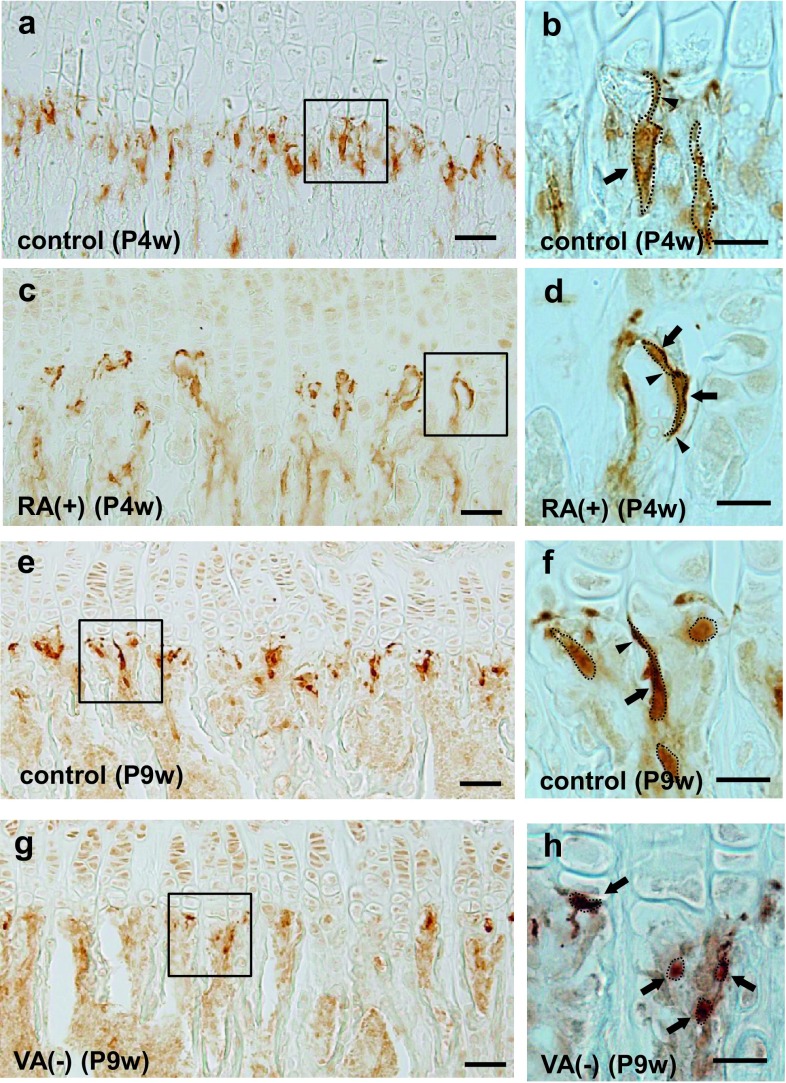
Chodro-osseous junction (COJ) of the growth plate in mouse tibiae. Light micrographs at lower (**a**, **c**, **e**, **g**) and higher (**b**, **d**, **f**, **h** *squares* in **a**, **c**, **e**, and **g**, respectively) magnifications at the COJ of the GP in mouse proximal tibiae of P4w control (**a**, **b**), P4w RA excess (**c**, **d**), P9w control (**e**, **f**), and P9w VA deficiency (**g**, **h**). *Dotted line* outline of each E-FABP-immunopositive septoclast; *arrows* septoclastic cell bodies; *arrowheads* septoclastic processes; *scale bars* 50 μm (**a**, **c**, **e**, **g**) or 20 μm (**b**, **d**, **f**, **h**)

In mice at P4w under normal physiological state, E-FABP-positive septoclasts were arranged longitudinally in the COJ at short intervals in the GP of the proximal tibiae (Fig. 2a), and their processes extended toward the GP cartilage (Fig. 2b) as shown in our previous study (Bando et al. 2014). Under RA excess, E-FABP-positive septoclasts were located in the COJ at longer intervals (Fig. 2c) and possessed shorter processes without reaching up to the GP cartilage (Fig. 2d) than those of the control (Fig. 2a, b). At P9w under normal physiological state, the distribution and shape of E-FABP-positive septoclasts were very similar (Fig. 2e, f) to those of P4w normal mice (Fig. 2a, b). Under VA deficiency, E-FABP-positive septoclasts were arranged at longer intervals (Fig. 2g), and became smaller in size (Fig. 2h) than those of the control (Fig. 2e, f) with their processes being indistinct (Fig. 2h).

**Fig. 3:**
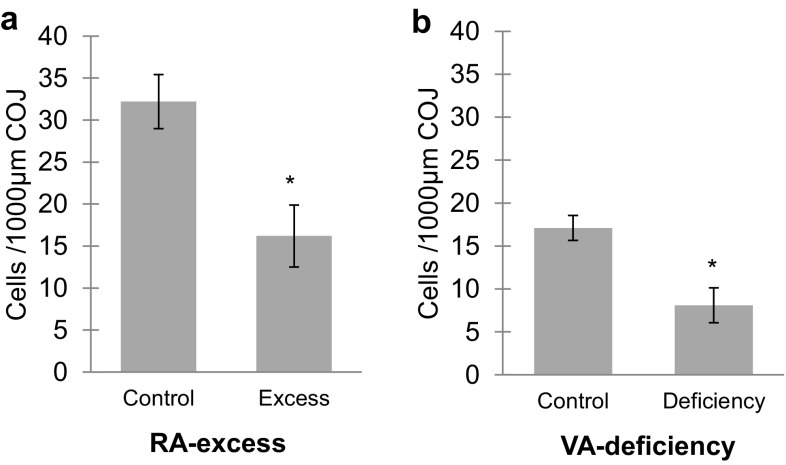
Graph showing numerical changes of E-FABP-immunoreactive septoclasts. The cells were counted at the COJ in an area of 1000-μm width ×200-μm height in the GP of mouse proximal tibiae of RA excess (**a**) or VA deficiency (**b**). Values are shown as mean ± SD; *P < 0.01 (*n* = 10)

Under RA excess, the number of E-FABP-immunopositive septoclasts per unit area in the COJ of proximal tibiae significantly decreased in proportion to the concentration of RA (Fig. 3a). Approximately 50% reduction of the cell number was induced by the oral administration of RA in comparison with those of the control. Such a remarkable reduction of the cell number as approximately 50% was also induced by VA deficiency (Fig. 3b).

## 3D morphological changes of septoclasts

**Fig. 4:**
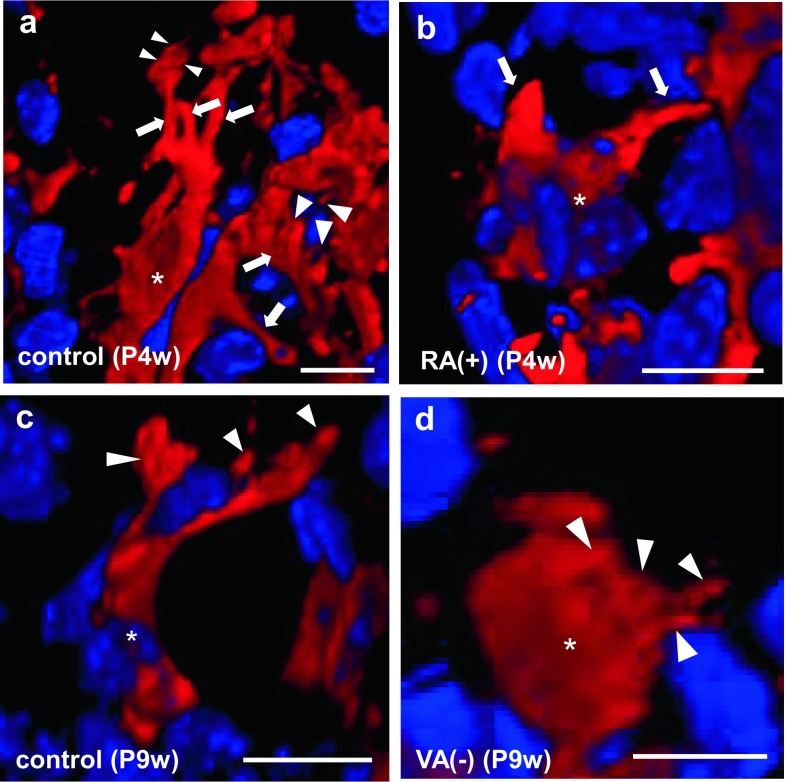
Three-dimensional (3D) images of E-FABP-immunoreactive septoclasts. Confocal laser-scanning microscopy of the 3D reconstruction of E-FABP-immunoreactive (*red*) septoclasts (**a**–**d**) at the COJ of the GP in mouse proximal tibiae of P4w control (**a**), P4w RA excess (**b**), P9w control (**c**), and P9w VA deficiency (**d**). Nuclei are stained with DAPI (*bluish purple*). *Asterisks* septoclastic bodies, *arrows* septoclastic processes, *arrowheads* microvilli (**a**) or short cytoplasmic spines (**d**); *scale bars* 10 μm

E-FABP-positive septoclasts of P4w normal mice had spindle-like cell bodies with several processes, and several microvilli were observed in the apex of their process (Fig. 4a) as we previously reported (Bando et al. 2014). The cell bodies of septoclasts under RA excess were spindle-like in shape but smaller in size (Fig. 4b) than those of the control (Fig. 4a). Their processes apparently decreased in number and shortened (Fig. 4b) compared with those of the control (Fig. 4a), and microvilli were not observed at process endings (Fig. 4b). The number of cell processes and microvilli of septoclasts at P9w (Fig. 4c) was less than that at P4w (Fig. 4a). The cell bodies of septoclasts of VA-deficient mice were spherical in shape and smaller in size (Fig. 4d) than those of the control (Fig. 4c). Short spines protruding from the cell body are observed, while cell processes were rarely found (Fig. 4d).

### Ultrastructural change of septoclastic processes

**Fig. 5:**
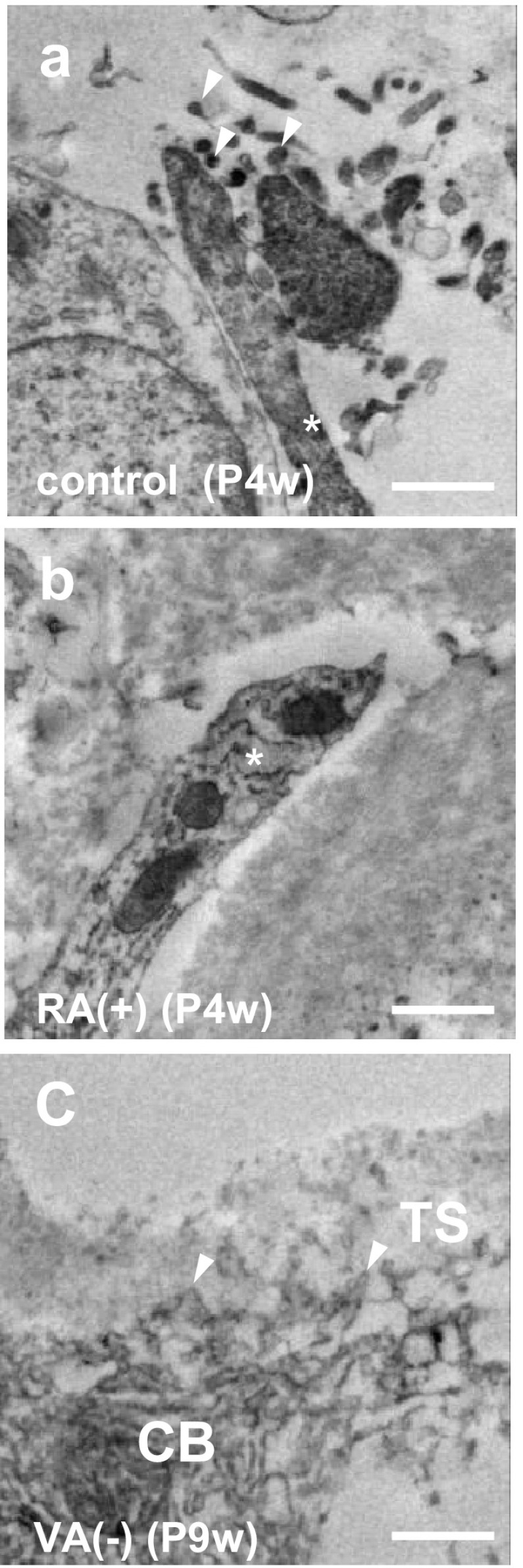
Process apex of septoclasts. Immunoelectron micrographs of E-FABP-immunoreactive septoclasts at the COJ of the GP of mouse proximal tibiae of P4w control (**a**), P4w RA excess (**b**), and P9w VA deficiency (**c**). *Asterisks* septoclastic processes; *arrowheads*: microvilli (**a**) or short spines of septoclastic cell body (**c**); *CB* septoclastic cell body, *TS* transverse septum; *scale bars* 1 μm

Under a normal physiological state at P4w and P9w, many microvilli were observed in the apexes of septoclastic processes (Fig. 5a). Processes of septoclasts under RA excess often penetrated into the cartilage matrices of the GP and the surface of their apex was smooth without microvilli (Fig. 5b). Septoclastic cell bodies with short spines under VA deficiency frequently attached to the transverse septa (Fig. 5c). These findings on E-FABP-positive septoclasts were in accordance with those by 3D morphology (Fig. 4).

### Localization of PPARβ/δ in septoclasts

**Fig. 6:**
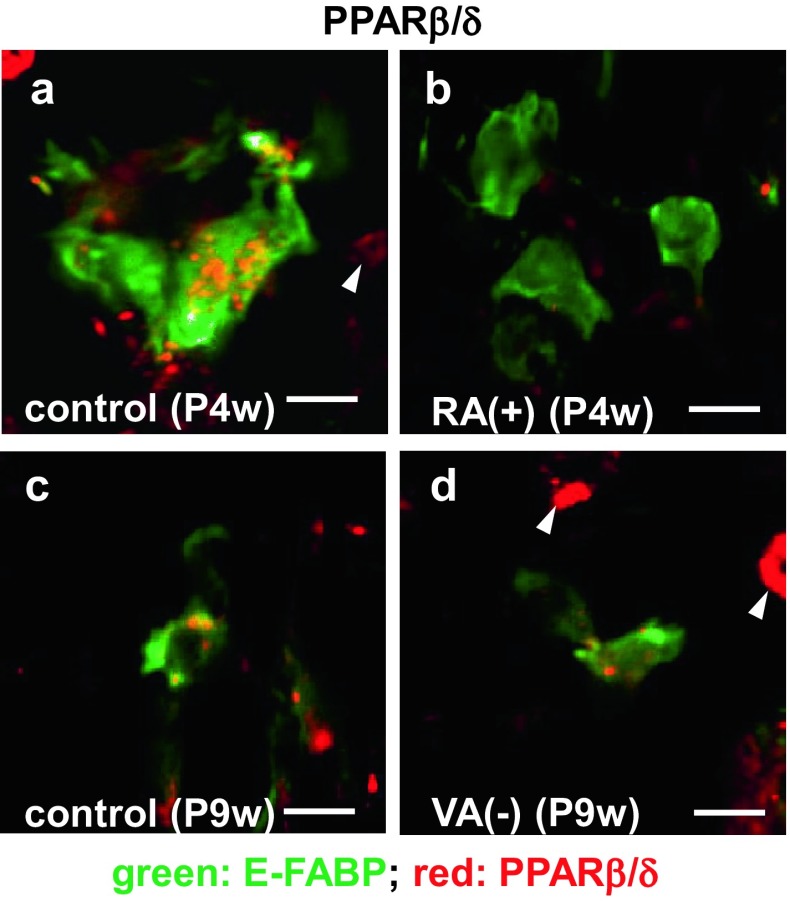
Double staining for E-FABP plus PPARβ/δ in septoclasts. Confocal laser-scanning micrographs of double immunostaining for E-FABP (*green*) plus PPARβ/δ (*red*) at the COJ of the GP in mouse proximal tibiae of P4w control (**a**), P4w RA excess (**b**), P9w control (**c**), and P9w VA deficiency (**d**). *Arrowheads* PPARβ/δ-immunoreactive chondrocytes; *scale bars* 10 μm

The immunoreactivity for PPARβ/δ was intense in the cytoplasm of E-FABP-positive septoclasts in the GP of P4w normal mice (Fig. 6a), whereas it was not detected in the cells under RA excess (Fig. 6b). The immunoreactivity in the cells of both P9w normal (Fig. 6c) and P9w VA-deficient mice (Fig. 6d) was weaker than that of normal P4w (Fig. 6a).

### Cell-death induction in septoclasts by RA excess

**Fig. 7:**
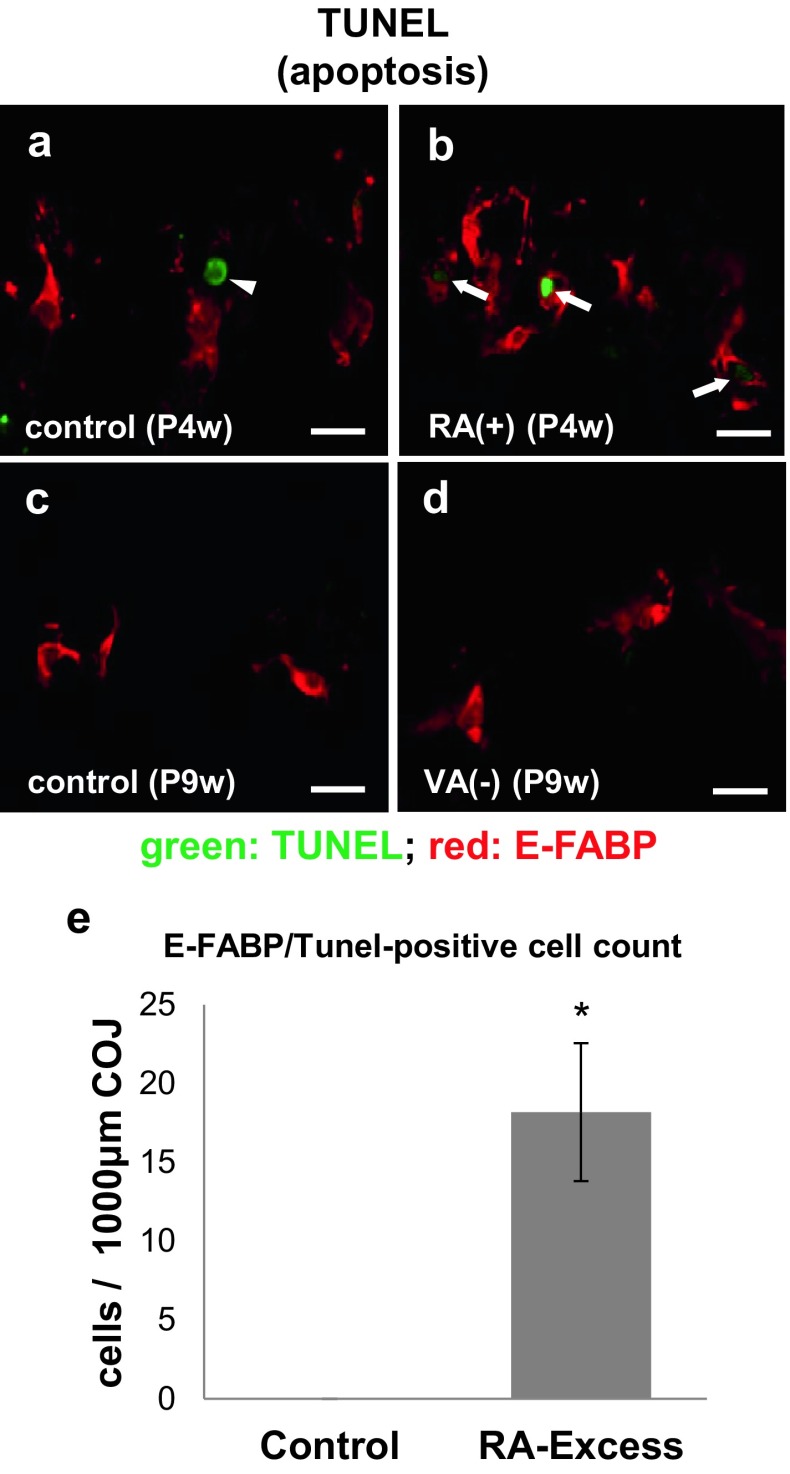
E-FABP-immunoreactivity and apoptosis in septoclasts. Confocal laser-scanning micrographs of double staining for E-FABP (*red*) plus TUNEL reaction (*green*) at the COJ of the GP in mouse proximal tibiae of P4w control (**a**), P4w RA excess (**b**), P9w control (**c**), and P9w VA deficiency (**d**) and the graph (**e**) showing the significant increase of simultaneously E-FABP-immunoreactive and TUNEL-positive apoptotic cells at the COJ of the GP in mouse proximal tibiae of RA excess in an area of 1000-μm width ×200-μm height as compared to the control. Arrows: TUNEL-positive septoclasts; arrow head: TUNEL-positive chondrocyte; *scale bars* 20 μm; mean ± SD; *P < 0.01 (*n* = 10)

To clarify the cause of decrease in the number of E-FABP-immunopositive septoclasts under RA excess and VA deficiency, we examined whether the cell-death was induced or not by these treatments. E-FABP-positive septoclasts simultaneously showing TUNEL-positive reaction representing the apoptosis were found exclusively under RA excess, whereas no apoptotic cells were detected in VA deficiency and the controls (Fig. 7a–d). In contrast, LC3-immunoreactivity representing the autophagy was not detected in E-FABP-positive septoclasts under RA excess, VA deficiency, or their individual controls (data not shown). In morphometrical analysis, a significant increase in the number of both E-FABP-immunoreactive and TUNEL-positive cells per unit area was seen under RA excess (Fig. 7e).

**Fig. 8:**
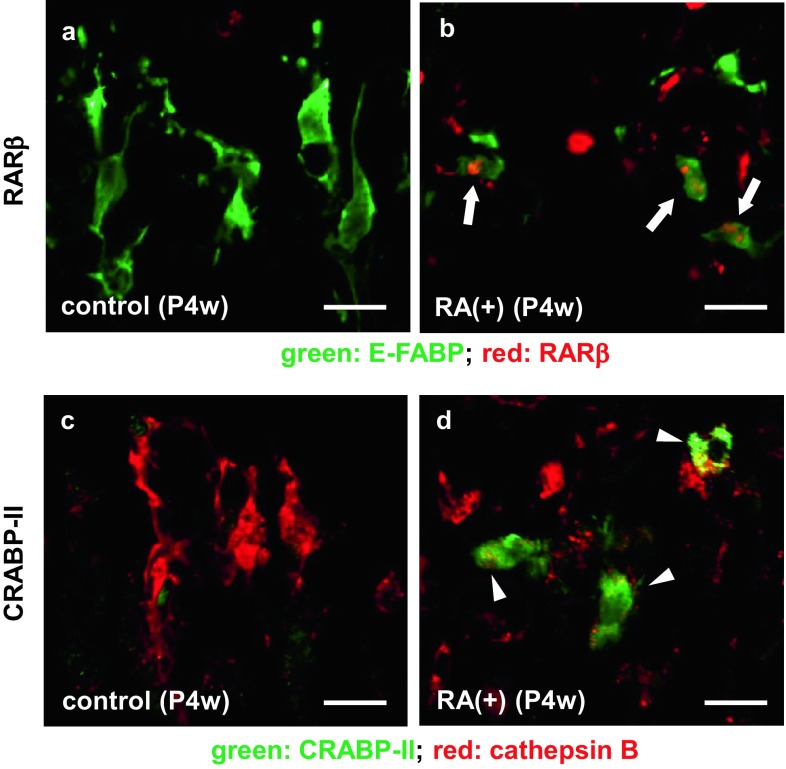
Double staining for E-FABP plus RARβ (**a**, **b**) and for CRABP-II plus cathepsin B (**c**, **d**). Confocal laser-scanning micrographs of double staining for E-FABP (*green*, **a**, **b**) plus RARβ (*red*, **a**, **b**), and for CRABP-II (*green* **c**, **d**) plus cathepsin B (*red* **c**, **d**) at the COJ of the GP in mouse proximal tibiae of P4w control (**a**, **c**) and P4w RA excess (**b**, **d**). *Arrows* RAR-immunoreactive septoclasts; *arrowheads* CRABP-II-immunoreactive septoclasts; *scale bars* 20 μm

The immunoreactivity for RARβ was detected in almost all E-FABP-immunopositive septoclasts, while that for CRABP-II was detected randomly in some of the septoclasts under RA excess. No immunoreactivity for either of the two molecules was found in E-FABP-positive septoclasts in control mice (Fig. 8a–d). The immunoreactivity for RARβ without E-FABP- immunoreactivity was also observed in chondrocytes (Fig. 8b).

### CD-RAP induction in E-FABP-positive septoclasts by VA deficiency

**Fig. 9:**
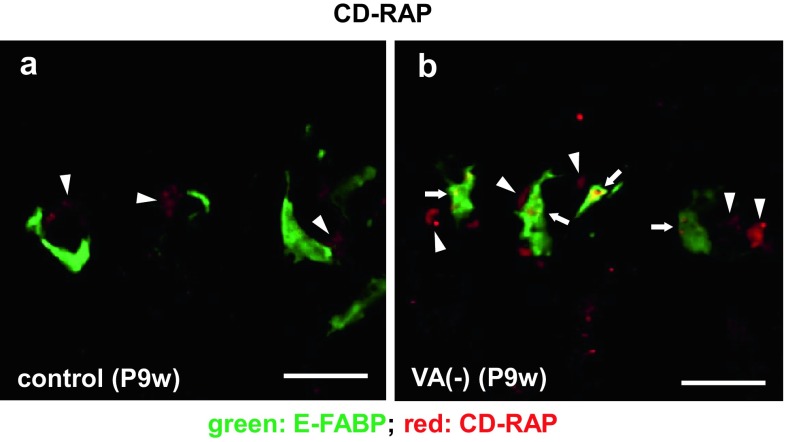
Double staining for E-FABP plus CD-RAP in septoclasts. Confocal laser-scanning micrographs of double staining for E-FABP (*green*) plus CD-RAP (*red*) at the COJ of the GP in mouse proximal tibiae of P9w control (**a**) and P9w VA deficiency (**b**). *Arrows* CD-RAP-immunoreactive septoclasts; *arrowheads* CD-RAP-immunoreactivity in chondrocytes; *scale bars* 20 μm

In the present normal mouse specimens, the immunoreactivity for CD-RAP was not found (Fig. 9a), whereas it was detected in the cytoplasm of E-FABP-positive septoclasts under VA deficiency (Fig. 9b).

## Discussion

Major findings of the present study are the decrease in number of E-FABP-positive septoclasts being simultaneously immunopositive for PPARβ/δ, and the increase in number of E-FABP-positive septoclasts showing TUNEL-positive apoptosis or immunoreactivities for RARβ and CRABP-II by means of administration of mice under RA excess. In general, RA as well as FAs is known to be an agonist of PPARβ/δ, and to induce the cell survival/proliferation via a signaling pathway mediated by E-FABP and PPARβ/δ (Schug et al. 2007). Those authors have proposed a model outlining the dual transcriptional activity of RA, that is, CRABP-II and E-FABP target RA to RAR and PPARβ/δ, respectively. The E-FABP/CRABP-II ratio determines whether to activate E-FABP/PPARβ/δ or CRABP-II/RARβ (Schug et al. 2007) and activated CRABP-II/RARβ pathway can interfere with E-FABP/PPARβ/δ pathway accompanied by down-regulation of PPARβ/δ (Yu et al. 2012). According to the model, RA possibly binds to CRABP-II to activate RARβ and to reduce the expression of PPARβ/δ in septoclasts under RA excess. Considering these views, the present findings suggest that the overdose of RA down-regulates the expression of PPARβ/δ, resulting in inhibition of the cell survival/proliferation signaling mediated by PPARβ/δ, and subsequently induces apoptosis of septoclasts via the cell-death-signaling mediated by RARβ activated by RA binding to CRABP-II. As a consequence, septoclasts decrease in number. In this regard, both E-FABP and PPARβ/δ have already been shown to be co-expressed in septoclasts under the physiological state (Bando et al. 2014).

Another finding of the present study was a significantly less number of septoclasts in mice fed by VA-free diet from P3w to P9w than normal control, and an induction of the immunoreactivity for CD-RAP in such decreased septoclasts. Considering the function of CD-RAP as a cell growth inhibitory factor without cytotoxicity (Blesch et al. 1994) and its strong expression in bovine articular cartilage cells under extremely low RA (Dietz and Sandell 1996), the enhanced decrease in number of septoclasts under VA-deficient condition is presumably caused by growth inhibitory effects of CD-RAP owing to the extremely low concentration of RA. Since the expression of PPARβ/δ was maintained under VA deficiency in the present study, the decreased amount of RA under VA deficiency possibly weakens the PPARβ/δ-mediated cell survival/proliferative effect, resulting in the enhanced decrease in number of septoclasts under the VA-deficient condition.

The processes of septoclasts extend toward the GP attaching to the uncalcified transverse septa, and the microvilli of the process apex penetrate into the transverse septa. These features are similar to the ruffled border of osteoclasts (Schenk et al. 1967; Lee et al. 1995; Nakamura and Ozawa 1996). The resorption of mineralized bone and/or calcified cartilage by osteoclasts/chondroclasts is performed in restricted regions of the ruffled border by hydrochloric acid and proteinases (Schenk et al. 1967; Vaananen et al. 2000). Septoclasts play roles to degrade uncalcified cartilage matrices of the GP using their long processes with developed microvilli and to digest them by endogenous enzymes such as cathepsin B (Lee et al. 1995) or MMP-13 (Nakamura et al. 2004).

In the present study, the septoclasts under RA excess were revealed to have fewer and shorter processes lacking in microvilli. In this regard, it is to be noted that osteoclasts of mouse osteosclerosis (*oc*) are characterized by poor development or absence of ruffled borders, ruffled borders forming bulbous expansions, and a marked reduction of lysosomal enzymes associated with the bone resorption, which is interpreted as a morphological evidence of decreased bone resorption (Seifert and Marks. 1985). It is, thus, suggested that from the present changes in morphology of septoclasts, the RA overdose down-regulates the cartilage resorption of septoclasts by stimulation of the RARβ signaling and induction of both apoptosis and morphological changes (Seewaldt et al. 1995; Lee et al. 2012). Judging from severe shortening or absolute absence of septoclastic processes, and short spines arising from their cell bodies and attaching to the cartilage matrix in VA-deficient specimens, the activity of cartilage resorption by septoclasts seems to be severely down-regulated. Because no expression of RARβ was found in atrophied septoclasts under VA deficiency, it is unlikely that the RARβ-mediated signaling is responsible for the apoptosis and morphological changes of septoclasts.

However, the present septoclasts under VA deficiency were found to induce the expression of CD-RAP, which is similar to the previous report of melanoma cells in terms of association with such morphological changes as seen in the septoclasts (Blesch et al. 1994). It is, thus, possible that the CD-RAP is responsible for the decrease in cell number and morphological changes of septoclasts. It is also suggested that the morphological changes of processes and their microvilli lead septoclasts to diminish the mechanical linkage between the cells and the transverse septa, resulting in reduction of the cartilage matrix resorption of septoclasts and induction of abnormal bone trabeculae including vertically arranged chondrocytes in their core under RA excess or VA deficiency. Induction of thinner GP by RA excess or VA deficiency was suggested to be induced by the inhibition of chondrocyte proliferation (Wolbach 1947; Frandsen and Becks 1962; De Luca et al. 2000), but abnormal bone trabeculae including chondrocytes are possibly formed by the failure in resorbing transverse septa by fewer and such atrophied septoclasts.

The present study successfully showed septoclasts to be negatively affected by RA in terms of the cell number and morphology, which is essential for the resorption of the transverse septa of the GP. The longitudinal growth of the long bone is attributed to proliferation of chondrocytes in the zone of proliferation in the GP, cartilage resorption by septoclasts and osteoclasts, and osteogenesis by osteoblasts (Mackie et al. 2011). The growth inhibition of longitudinal bones induced by excessive intake or deficiency of VA (Pease 1962; Wolbach 1947; Frandsen and Becks 1962) is presumably associated with a reduction of septoclastic cartilage resorption regulated by RA, and the appropriate intake of VA may be necessary for the bone growth to be maintained by normal functional exertion of septoclasts.

Altogether, the present findings support the hypothesis that RA incorporated into septoclast during the cartilage resorption regulates the growth and/or death of septoclasts using the signaling pathways mediated by E-FABP and PPARβ/δ.
